# Tunable high-order harmonic generation in GeSbTe nano-films

**DOI:** 10.1515/nanoph-2023-0859

**Published:** 2024-04-18

**Authors:** Viacheslav Korolev, Artem D. Sinelnik, Mikhail V. Rybin, Petr Lazarenko, Olga M. Kushchenko, Victoria Glukhenkaya, Sergey Kozyukhin, Michael Zuerch, Christian Spielmann, Thomas Pertsch, Isabelle Staude, Daniil Kartashov

**Affiliations:** 9378 Friedrich-Schiller University Jena, Jena, Germany; 65071 ITMO University, St. Petersburg, Russia; Ioffe Institute, St. Petersburg, Russia; 386236 National Research University of Electronic Technology, Moscow, Russia; 201631 N.S. Kurnakov Institute of General and Inorganic Chemistry, Moscow, Russia; University of California at Berkeley, Berkeley, USA; Fraunhofer-Institute for Applied Optics and Precision Engineering IOF, Jena, Germany; Max Planck School of Photonics, Jena, Germany

**Keywords:** high harmonic generation, GST, active nonlinear photonics, phase change material

## Abstract

High-order harmonic generation (HHG) in solids opens new frontiers in ultrafast spectroscopy of carrier and field dynamics in condensed matter, picometer resolution structural lattice characterization and designing compact platforms for attosecond pulse sources. Nanoscale structuring of solid surfaces provides a powerful tool for controlling the spatial characteristics and efficiency of the harmonic emission. Here we study HHG in a prototypical phase-change material Ge_2_Sb_2_Te_5_ (GST). In this material the crystal phase can be reversibly changed between a crystalline and amorphous phase by light or electric current mediated methods. We show that optical phase-switching is fully reversible and allows for dynamic control of harmonic emission. This introduces GST as new addition to materials that enable flexible metasurfaces and photonic structures that can be integrated in devices and allow for ultrafast optical control.

## 1 Introduction

The process of high-order harmonic generation (HHG) was first observed in gases more than three decades ago [1] and nowadays it is regarded as one of the fundamental strong-field phenomena and lies at the heart of attosecond physics [2]. Later, this effect was discovered in solids, both in crystalline [3], [4], [5], [6], [7], [8] and amorphous [9], [10] states. Except of its importance for the fundamental physics of laser–matter interaction, HHG in solids is considered as a powerful tool for a new type of ultrafast spectroscopy in solids [11], [12], [13], [14], [15], [16] and perspective platform for design of compact sources of attosecond pulses [17], [18], [19], [20].

**Figure 1:**
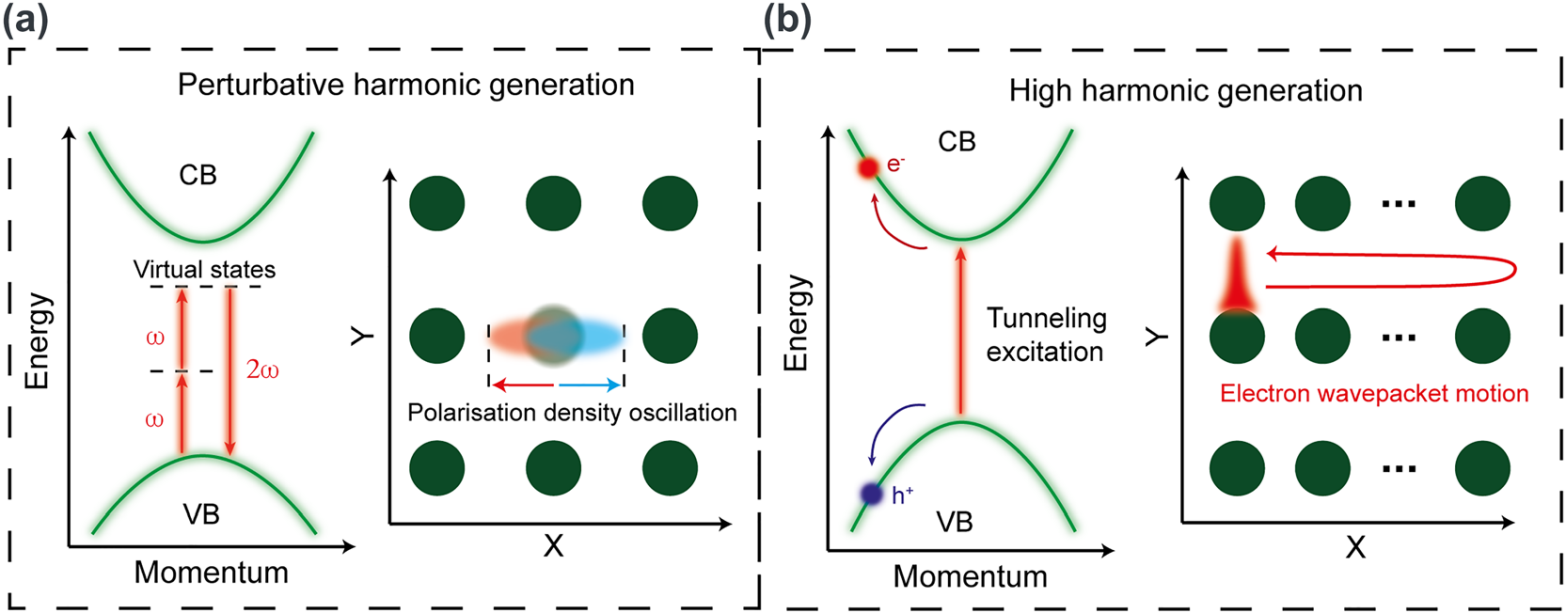
Comparison of harmonic generation in the perturbative and strong field regimes. (a) An example of the perturbative second harmonic generation, explained by virtual transitions in the energy domain and by nonlinear polarization of bound electrons localized at the lattice atoms. (b) HHG as a result of multiphoton/tunnel excitation to the conduction band and anharmonic electron/hole motion in the conduction/valence bands. In real space electron wavepacket traverses many lattice constants when moving within the optical cycle.

The principal difference between low-order (second, third) harmonic generation in conventional, weak field nonlinear optics and strong field process of HHG is the nonlocal nature of the last. This is schematically shown in Figure 1. In the perturbative nonlinear optics, harmonic generation can be understood as a virtual transition from the valence band with absorption of two or three pump photons and emission of the harmonic quanta (Figure 1(a)). In the real crystal lattice space, the harmonic emission originates from the nonlinear polarization induced by the anharmonic oscillations of the electron charge density localized near the lattice atoms. In contrast, in strong filed regime electron is excited via multiphoton absorption or tunnelling to the conduction band. As a result, coherent electron/hole wavepackets are created both in the energy-momentum and real space domains. The wavepackets in the valence/conduction bands are driven by the strong laser field far away from the minimum gap excitation point, traversing large part or even entire Brillouin zone. The corresponding electron/hole wavepackets in the real space oscillate in the laser field with the quiver motion covering many lattice constants (Figure 1(b)). This nonlocal nonlinear dynamics in the energy-momentum and real space results in two sources of harmonic emission: the intraband nonlinear current, related to the non-parabolicity of the bands (or anharmonicity of the lattice potential, in which electron is driven through the crystal by the laser field), and interband nonlinear polarization. The last is the direct analogy of HHG mechanism in gases and linked to the phase-locked oscillations of electron-hole pairs within the optical cycle and recombination when they meet in the crystal [3], [5], [7]. It is also very sensitive to any scattering processes that can cause a decoherence in the field driven electron-hole motion, in contrast to the first mechanism that is rather robust against the particle interactions.

Optimization of the efficiency of energy conversion from the driving laser field to the emitted harmonics is one of the fundamental problems in nonlinear optics. Different methods of nanophotonics were suggested to control and optimize the HHG process [21], [22], [23], [24]. Integrated nanophotonic structures are used to tailor far field spatial profile of vacuum ultraviolet (VUV) emission sources based on HHG in solids [21], [25]. Plasmonic field enhancement in metal nanoparticles [26], [27] and an all-dielectric meta-optical platform [28] exploiting Mie-type nanoparticles as resonators for the field localization have successfully been applied to increase the HHG yield. In particular, metasurfaces of resonant silicon nanoparticles are promising to significantly increase the intensity of HHG due to localization of the electromagnetic modes at the pumping wavelength [29]. However, the fabrication of meta-optical devices requires high precision of the technological processes, and the designed platforms are lacking tunability, which limits their application for active nanophotonics.

Finally, methods of adaptive control over resonance properties of metasurfaces by ultrafast optical changes in the index of refraction, demonstrated recently in the low field regime [30], [31], [32] are not efficient for HHG. This is because HHG intrinsically implies generation of high-density electron-hole plasma as the first step of strong field excitation. Therefore, dynamically changing during the laser pulse duration plasma density will lead to drastic changes in the resonant properties, as it is shown in [23].

Recently, phase change materials (PCM) such as Ge-Sb-Te, Ge-Sb-Se-Te and Sb-Se attracted a lot of interest of the photonic community because of a non-volatile and reversible phase transition between two metastable crystalline and amorphous states [33], [34], [35], [36], [37]. These materials exhibit dramatic changes of their optical and electrical properties as a result of the phase transition. Both amorphous-to-crystalline and inverse transitions can be triggered optically by laser pulses depending on duration and fluence. This unique feature of PCM allows for implementing switchable metasurfaces [38], [39], rewritable metalens [39] and tunable periodic structures [40], [41]. In most studies, PCM is used as a passive substrate affecting the resonant properties of the structural elements made of a different material. In particular, a GST sublayer was deposited for the control of the second and third harmonics generation from silicon metasurfaces [42] or gold structures [43], [44]. Recently, Mie-type PCM nanoparticles were shown to provide twofold functionality as a cavity for field enhancement and as a source of second harmonic radiation [45]. The second harmonic signal shows amplification by an order of magnitude when the phase transition in GST occurs.

Here we report on observation of high-order harmonic generation in GST nanofilms and demonstrate strong modulation in harmonic intensities by all-optical reversible switching between the amorphous and crystalline phases. It is worth noting that GST, unlike other PCMs, is a stable compound and does not degrade during the phase transition, so no additional protective layers were used. We show up to tenfold enhancement in fifth and seventh harmonics yield due to switching from amorphous to crystalline phase, whereas the ninth harmonic was detected in the crystalline GST only. Also, we demonstrate that the dependence of harmonics yield on the laser intensity for both phases shows the threshold-like behaviour at low pump intensities that might be attributed to scattering of carriers on defects.

## 2 Results and discussion

**Figure 2:**
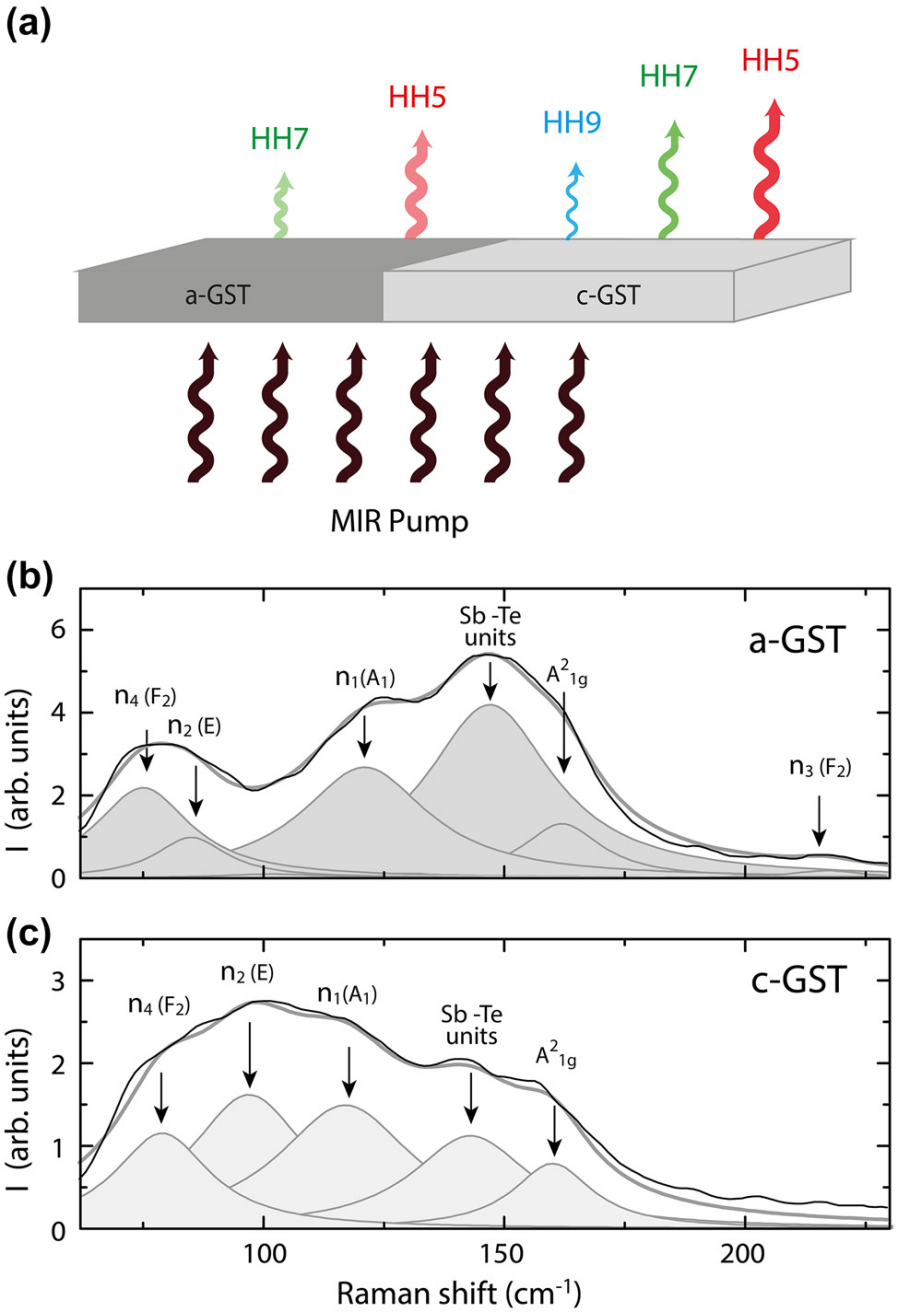
Schematic of harmonic generation and Raman spectra. (a) Schematic of harmonic generation from amorphous (a-GST) and crystalline (c-GST) GeSbTe nanofilm. Raman spectra from the amorphous (b) and crystalline (c) phases of GST (black curves) with vibration mode decomposition (grey curves).

Figure 2(a) shows a schematic representation of HHG in a Ge_2_Sb_2_Te_5_ (GST) nanofilm for different phases. Thin film of GST composition with thickness of 20 nm was deposited on a sapphire substrate. Using DC magnetron sputtering we produced our polycrystalline films for the following parameters: pressure in the chamber 3.0 × 10^−3^ Pa, Ar pressure 5.7 × 10^−1^ Pa, and for a DC power of 100 W. The initially sputtered GST film is in the amorphous phase. To switch the initial amorphous GST film into the crystalline state, we used a Ti:Sa laser (Avesta, Russia) with a wavelength of 790 nm, a pulse repetition rate of 80 MHz, and a pulse duration of 70 fs combined with direct laser writing system (LZH, Germany) to move the laser beam over the surface of the sample at a speed of 100 μm/s in the *X*–*Y* plane. The laser beam was focused by an Olympus objective 40× with a NA = 0.75 and the mean power density on the sample was 23 mW/μm^2^. The film was exposed to local heating by laser radiation. Raman spectra were measured for detecting the GST phase after nanofilm deposition and optical switching of the phase state. These results shown in Figure 1(b) and (c) confirm the phase transition from the amorphous to crystalline state in the nanofilm after optical irradiation. Decomposition of the spectra displays specific vibration modes of the Ge–Te and Sb–Te units. The peak at 147 cm^−1^ is likely due to the vibrations of the Sb–Te units, however other origin such as Sb–Te_3_ pyramidal structures or defective octahedral configuration of Sb atoms are also possible. The spectra comparison reveals that peaks change their spectral position and mutual intensity. The peaks become narrower after crystallization because of the lattice ordering and additional mechanical stress. The overall spectrum modification is in reliable agreement with reported data on switching between amorphous and crystalline cubic phases of GST [46]. A more detailed description of the Raman spectra decomposition before and after optical switching is presented in the Supplementary (Figure 1S).

**Figure 3:**
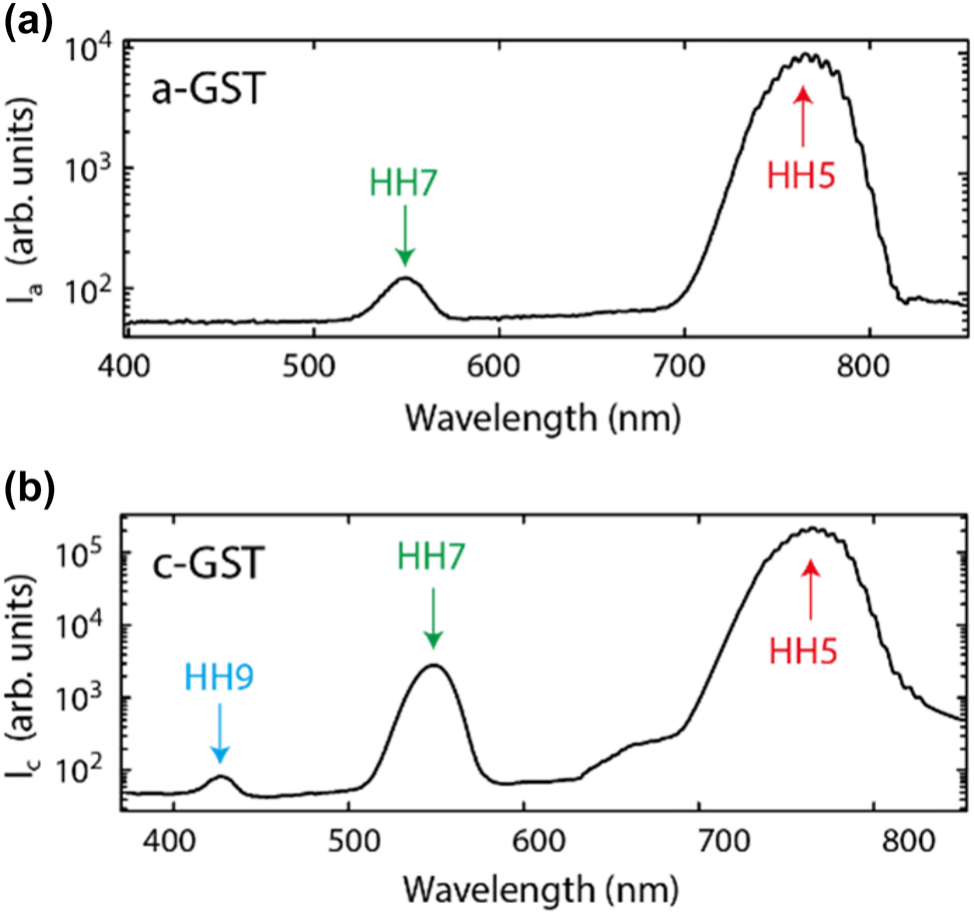
Measured HHG spectra for both GST phases. (a) HHG spectrum from the GST in amorphous phase. (b) HHG spectrum from the GST in crystalline phase. Note log-scale for the spectral intensity axes.

To generate high harmonics from the GST film, a femtosecond optical parametric amplifier (Light Conversion TOPAS Prime) was employed. The OPA was pumped by a 35 fs, 800 nm laser source (Coherent, Astrella), operating at a 1 kHz repetition rate. Signal and idler beams were mixed in a noncolinear difference frequency generator (NDFG) stage, providing an 80 fs mid-IR pulse of 3.8 µm wavelength. The duration of the mid-IR pulses was measured with a second harmonic-based frequency-resolved optical gating (FROG) setup. The pump pulses were focused onto the GST film with a 10 cm focal length CaF_2_ lens to a spot size of 1200 μm^2^ (40 µm diameter), measured by an IR CCD camera. The transmitted HHG signal was collected by an ×50 NA = 0.45 NUV Plan Apo objective lens and then focused on a slit of the spectrometer (Andor Kymera-328i) equipped with a cooled UV-enhanced CCD camera. For optical diagnostics of the film, on-site microscopy, consisting of a white light source and a colour CCD camera, was employed. The film was carefully checked before and after the measurements to rule out laser induced damage during the measurement. Figure 3 shows high-harmonic spectra produced in the GST nanofilm at an incident excitation intensity of 0.45 TW/cm^2^ (here and further in the text intensity value in vacuum is used). As can be seen in Figure 3(a), only the fifth and seventh harmonics are detected from the amorphous GST phase. In contrast, harmonics up to ninth order are detected in the HHG spectrum from the crystalline GST (Figure 3(b)). Besides, the fifth and seventh harmonics from the crystalline phase are at least one order of magnitude more intense than from the amorphous phase. In both phase state, spectral intensities of harmonics show an exponential decrease with the harmonic order.

The exponential decay in harmonic intensities with harmonic order suggests that the main mechanism of generation in both phase states is the nonlinear intra-band Bloch current [4], [47]. In this case the emitted spectrum has a plateau-like behavior extending up to harmonic order ∼*ω*_*B*_/*ω* and exponentially decaying for higher frequencies [4]. Here *ω*_*B*_ = *eE*_0_ *d*/*ℏ* is the Bloch frequency, *E*_0_ is the amplitude of the electric field in the laser pulse, *d* is the lattice constant and *ω* is the laser frequency. In our experiments, for the intensity 0.7 TW/cm^2^ we estimate the field amplitude *E*_0_ ≈ 0.14 V/Å (this includes ≈ 0.62 reduction of the field from the vacuum value inside the 20 nm film due to the reflection from the air and sapphire substrate interfaces) and, assuming 4.2 Å lattice constant of GST [36], the ratio *ω*_*B*_/*ω* ≈ 1.8. Therefore, it is expected that the intensities of harmonics starting from 3rd would drop exponentially as a function of the harmonic order.

**Figure 4:**
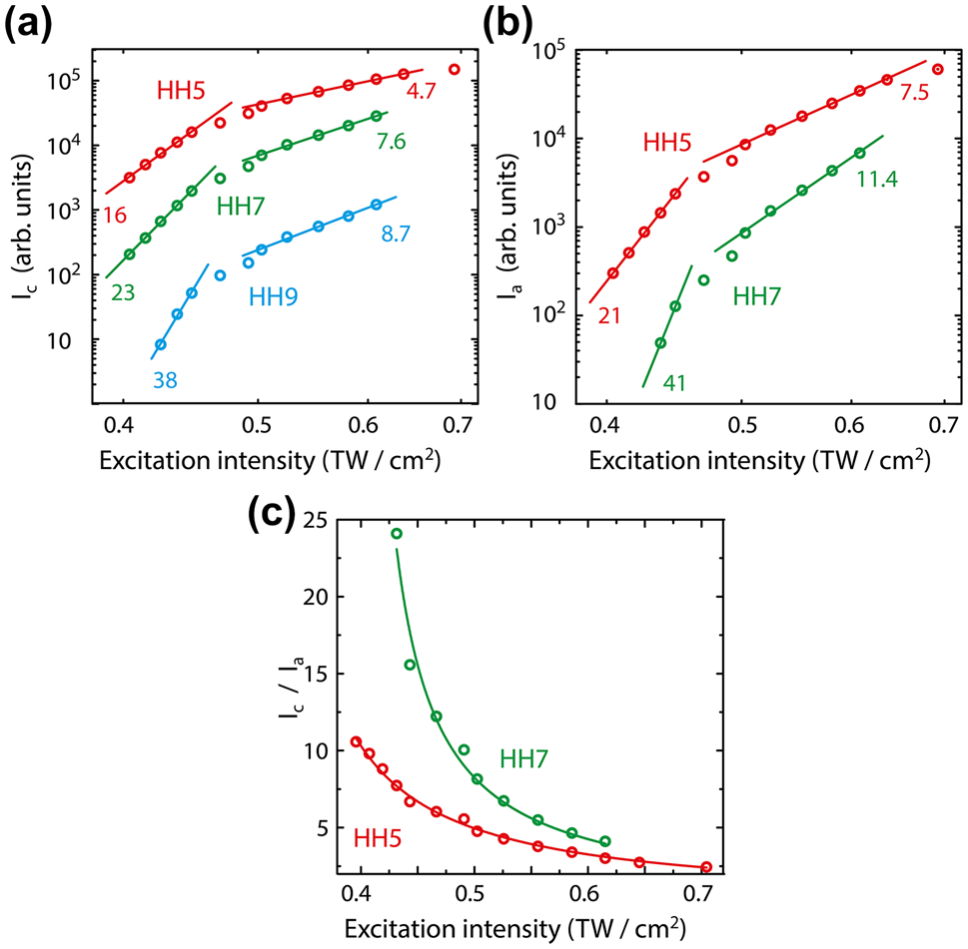
Intensity scaling of the harmonic intensities. Intensity of harmonics generated in the crystalline (a) and amorphous (b) GST nanofilm in dependence on the pump intensity. The red, green and blue circles show intensity of harmonic yield of 5th, 7th and 9th order, correspondingly. (c) The ratio of the intensities of harmonics, generated in crystalline and amorphous GST nanofilm in dependence on the pump intensity. The circles are measured values. The dashed lines are fittings by the reciprocal function.

Figure 4(a) and (b) show the scaling of the individual harmonic signals versus the fundamental excitation intensity for the two different phases. For both phases, the harmonic yields scale with a power index much higher than the corresponding harmonic’s order, if the excitation intensity is below ≈ 0.52 TW/cm^2^. For the pump intensity exceeding 0.52 TW/cm^2^ all curves exhibit power dependence with the power index close to the corresponding harmonic order. This scaling is very surprising, because for lower intensities the harmonic yield is given by the perturbative scaling (power index is equal to harmonic’s order). Only for higher intensities a deviation is expected, and the power index become independent on harmonic’s order, manifesting non-perturbative regime of generation [48]. Figure 4(c) displays the intensity ratio of the fifth and seventh harmonics generated in the GST nanofilm in amorphous and crystalline phases, respectively. These intensity ratios decrease with the pump intensity by a reciprocal law. At low pump intensities of 0.45 TW/cm^2^, the intensity of the seventh harmonic generated in the crystalline phase is 20 times to the amorphous one, whereas at higher pump intensity of 0.62 TW/cm^2^ the ratio is about 4.

**Figure 5:**
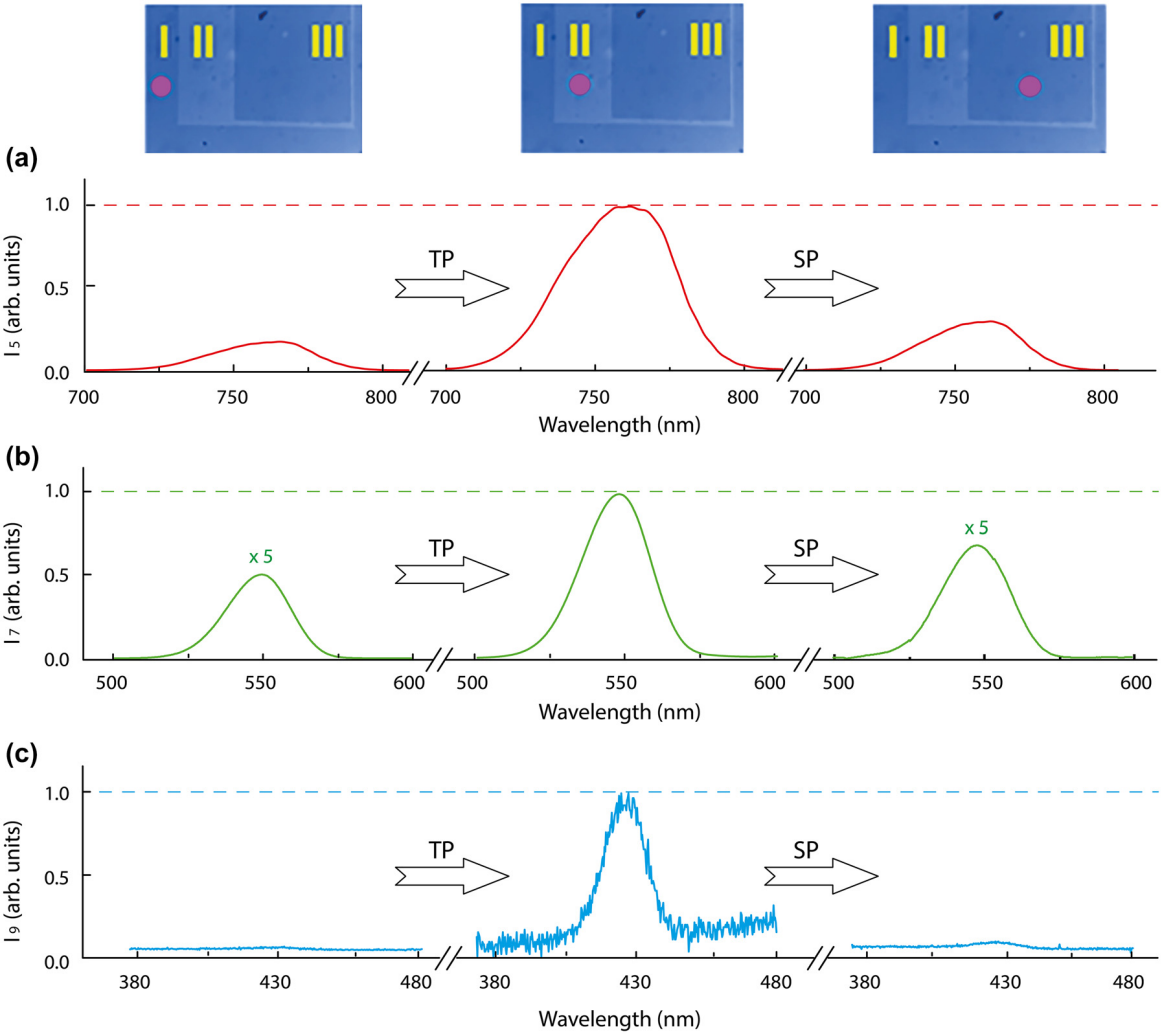
Reversible control over the intensity of high-order harmonics. (a) Fifth harmonic spectrum for the initial amorphous phase (initial a-GST), crystalline phase and after re-amorphization. (b) The same for 7th harmonic. (c) The same for 9th harmonic. Insets in the top show microscope images of the nanofilm with the original amorphous structure, partially crystallized and with partial re-amorphization of the crystalline region. Brighter area corresponds to the crystalline phase of GST. The circle marks the beam position on a sample.

A key feature of PCM is a possibility of a multicycle switching between amorphous and crystalline phases. The initial phase of amorphous GST nanofilm can be changed by optical absorption to crystalline and vice versa multiple times [43]. We examine the HHG in GST nanofilm undergoing several cycles of phase modification. As a proof of principle experiment, we do not change the phase *in-situ* by a control laser beam. Instead, a part of the initial GST nanofilm (after the GST deposition process, area I in Figure 5) was changed to the crystalline phase (area II in Figure 5) by a low intensity pulse train (TP) with wavelength 800 nm and repetition rate 80 MHz. Then a part of the crystalized film was converted back to an amorphous phase (area III in Figure 4) by applying a single laser pulse (SP) of high energy at the wavelength 1550 nm. Thus, the film was partitioned into three areas – initial amorphous, crystallized and re-crystallized back to amorphous – and comparative HHG spectra were measured by moving the pump spot across the nanofilm surface. Note, that due to low absorption at the mid-IR driving wavelength, used in our experiments, we did not observe re-crystallization of the film during HHG measurements for pump energies below 0.45 µJ and intensities below 1 TW/cm^2^. This is confirmed by microscopy diagnostics of the interaction region and by reproducibility of the HHG spectra when varying the pump intensity from high to low values.

The relative intensities of different harmonics for the fixed pump intensity 0.54 TW/cm^2^ and different GST phases are shown in Figure 4. Changing the phase from amorphous to crystalline results in a strong increase in the intensity of the 5th (≈5 times) and 7th (≈10 times) harmonics, and beside the 9th harmonic is detected in the spectrum. Re-amorphization of the crystalline film results in a decrease in the intensities of the 5th and 7th harmonics to their original values and the 9th harmonic is barely detected above the noise.

To understand the unusual, threshold-like dependence of high-order harmonic intensities on the pump intensity, we note that both amorphous and crystalline GST are indirect semiconductors [48]. The indirect bandgap is 0.5 eV and 0.7 eV in the crystalline and amorphous phase correspondingly [49], [50], [51], [52]. However, strong field excitation proceeds via a vertical transition to fulfil momentum conservation requirement. Also, the fact that harmonics with an energy ≥1.6 eV were observed suggest that transitions to higher laying conduction bands are involved as the first step of the generation process. It is worth noting that there are no fundamental restrictions on the generation of the 9th harmonic from GST in the amorphous phase, but this is only possible with an increase in the pump intensity. However, a further increase in the pump intensity leads to the destruction of the GST film. We measured the optical constants of crystalline and amorphous films, used in the experiments (see Figure 2S in Supplementary Materials). These measurements suggest that the direct optical bandgap is much higher and about 1.9 eV in crystalline and 3 eV in amorphous GST. The first value matches well the value of the direct bandgap in crystalline GST reported in Ref. [50]. Further, the results in Figure 3 can be compared to the results on the pump intensity dependence of harmonic’s yield in amorphous Si, measured under very similar experimental conditions and published earlier [10]. Amorphous and crystalline Si has an electronic structure similar to GST with the indirect bandgap ∼1 eV and direct bandgap 3–4 eV [53]. Harmonic yield, measured for the 3.8 µm driving laser wavelength and in the range of pump intensities 0.05–0.5 TW/cm^2^, shows power dependence on the pump intensity with the power index smaller than the corresponding harmonic’s order [10]. At high pump intensities, harmonics are generated in GST by analogy with Si.

Also, it is noteworthy that the efficiency of HHG from the GST film is similar to the efficiency of generation in a 1 µm thick Si film, used in our previous work [10]. Thus, we suggest that GST can be used for nanophotonics platforms as an alternative to Si-based technology with highly advantages possibility for easy all-optical control over the optical properties of the designed metasurfaces.

**Figure 6:**
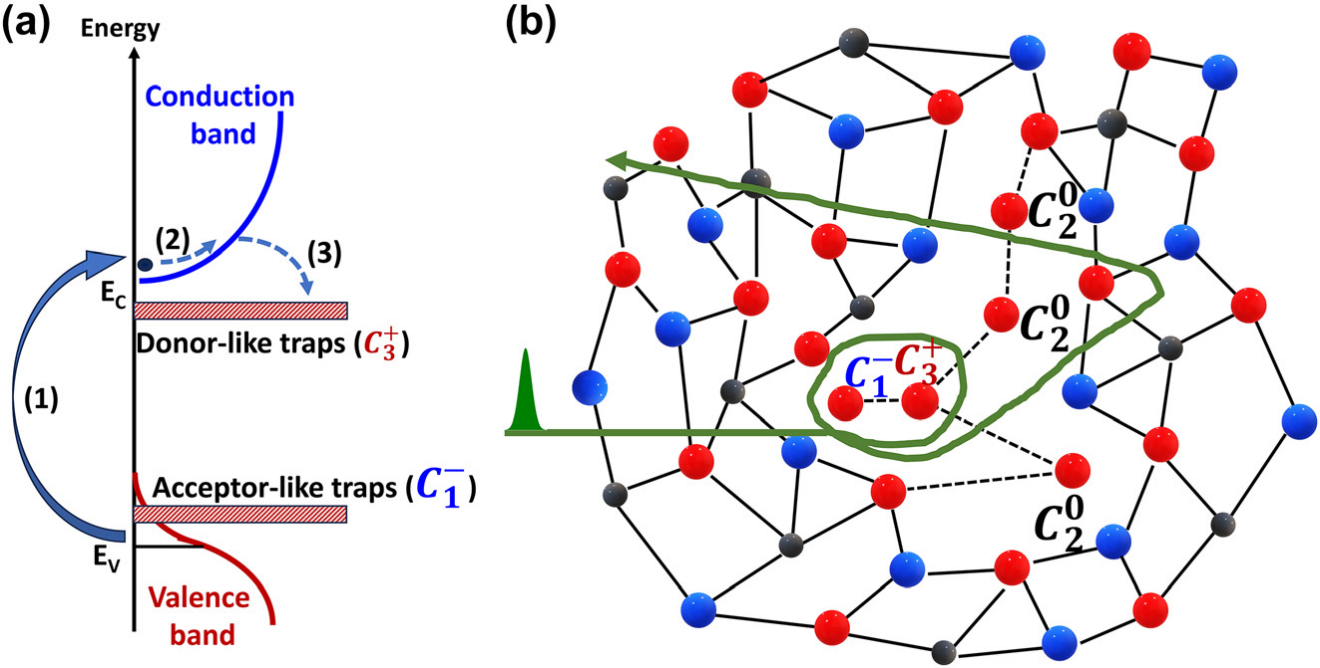
HHG suppression due to carrier scattering on charged defects. (a) Schematic energy diagram of amorphous GST. As the first step, electron is excited to the conduction band. Second step – intraband acceleration. Third step – electron capture on the donor-like trap state (hole capture on the acceptor-like trap state). (b) Schematic visualization of the plane projected crystal structure in amorphous GST (red balls are Te atoms, blue balls are Sb atoms, black balls are Ge atoms) with structural charged defects and quasi-trapped motion of the electron wavepacket (green line). $C_{2}^{0}$ is fundamental state, $C_{3}^{+}$ and $C_{1}^{-}$ is differently coordinated defect centers.

To explain the strikingly different, threshold-like dependence of harmonic yield at low pump intensities in GST, we suggest scattering of carriers on charged defect states, as it is illustrated in Figure 6. In contrast to Si, GST has in both phases very high density (>10^20^ cm^−3^) of acceptor-like traps and vacancies, located near the top of the valence and the bottom of the indirect conduction bands [49] (Figure 6(a)). Our assumption is that at low pump intensity the velocity of the electron wavepacket, defined by the laser vector potential, is relatively low and as a result of the scattering the electrons are trapped or quasi-trapped on the defects (Figure 6(b)). Following the detail analysis and nomenclature suggested in [49], we consider in Figure 6 charged and neutral defects, formed by valence-alternation pairs of Te atoms. Here *C* stands for chalcogen, the subscript is the covalent coordination number and superscript shows the charge state. The trapping leads to a dephasing between the driving laser field and the nonlinear current, thus significantly suppressing HHG efficiency. However, when laser intensity exceeds a certain critical value, scattering efficiency drops and power dependence in harmonic’s yield follows the perturbative scaling. The suggested crucial role of defects and trap states in HHG process can also explain order of magnitude difference in harmonic intensities between the crystalline and amorphous phases, mentioned above (Figure 2), because in the former phase the density of the defects and trap states is substantially higher [49].

## 3 Conclusions

In conclusion, we report on HHG in the phase change materials GST, demonstrating that the cyclic switching of the crystalline phases leads to strong modulation in harmonic intensities, essentially switching them reversibly on and off when changing from amorphous to the crystalline phase, and the modulation amplitude depends on the harmonic order. Our results suggest that application of PCM opens new perspectives in designing tunable and programmable metasurfaces and other active, switchable nanophotonics platforms for strong field physics applications, including compact attosecond pulse sources. For example, a PCM material can be used in nanoscale heterostructures with another semiconductor material like MgO or ZnO. In this case a large gap semiconductor is used as a HHG source, whereas PCM function is the tunability of the resonance properties of the metasurface. It is noteworthy that the efficiency of HHG in GST is similar to the efficiency of generation in Si. However, in contrast to Si-based photonic platforms, changes in linear and nonlinear optical properties of GST can be tuned continuously between the amorphous and crystalline phases, employing partial amorphization/crystallization of the material, and the switching can be done all optically, electrically or using temperature control.

## Supplementary Material

Supplementary Material Details
