# Supplementary material for: Tunable high-order harmonic generation in GeSbTe nano-films
Source: Nanophotonics. 2024 Apr 18;13(18):3411–9. doi: 10.1515/nanoph-2023-0859 (PMC11501657; doi:10.1515/nanoph-2023-0859)
Supplement: Supplementary file 1 — Supplementary Material Details [file j_nanoph-2023-0859_suppl_001.docx]

**Supplementary Information**

High-order harmonic generation in phase change materials

Viacheslav Korolev, Artem D. Sinelnik*, Mikhail V. Rybin, Peter Lazarenko, Olga M. Kushchenko, Victoria Glukhenkaya, Sergey Kozyukhin, Michael Zuerch, Christian Spielmann, Thomas Pertsch, Isabelle Staude, Daniil Kartashov


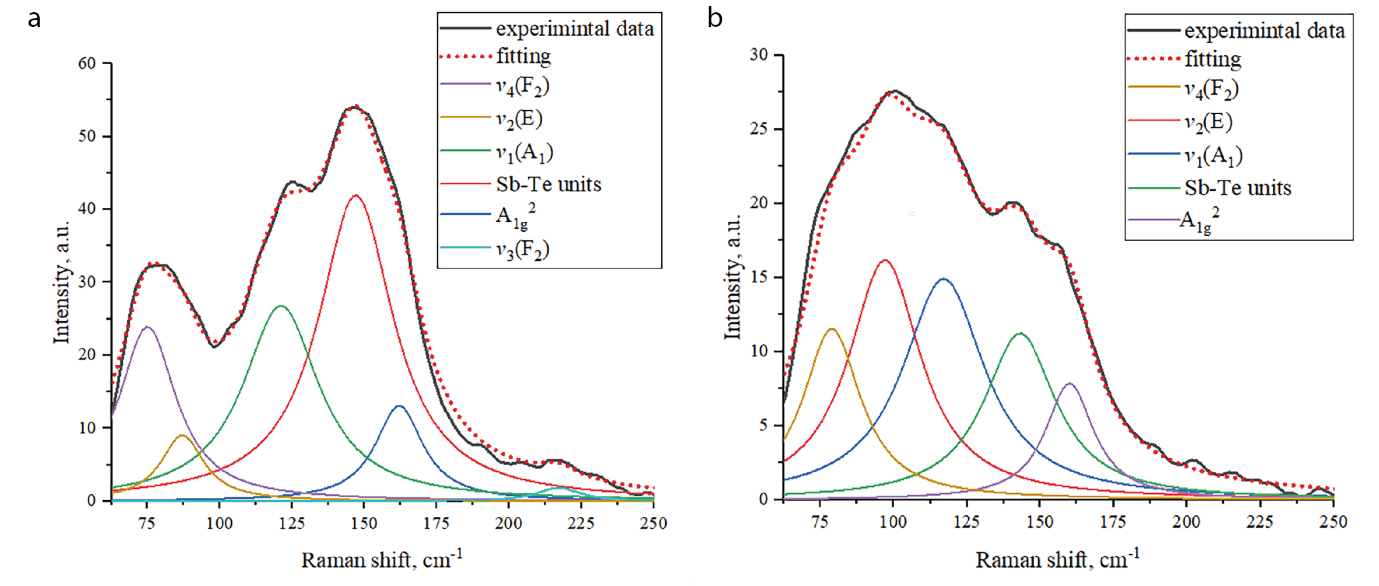


**Figure S1:** Raman spectra a) amorphous state and b) crystalline state

Raman spectra were measured for detecting the GST phase after nanofilm deposition and optical switching of the phase state. We acquired Raman spectra using excitation laser with 514 nm wavelength and a laser spot of 4 μm in diameter. The excitation power was limited to 0.1 mW to avoid phase transformation by heating effect during the acquisition of spectra. The deconvolution of Raman spectra was carried out using Lorentzian fitting. We identified 6 peaks at 75, 87, 121, 147, 162 and 217 cm^-1^ in the spectrum of initial films. Peaks with maximum at 75, 87, 121 and 217 cm^-1^ are associated with Ge-Te units,[1-6] peaks at 147 cm^-1^ and 162 cm^-1^ can be explained by vibrations of Sb-Te units.[1,5-9] It should be noted, that the band at 147 cm^-1^ can include vibrations of one or few structural units such as pyramidal units of Sb-Te3[3, 9] and defective octahedral configurations of Sb atoms.[5, 10] We did not assign specific vibrational modes to this band because of the proximity of the location of peaks position and the difficulty of their unambiguous and error-free decomposition. It can be easily seen that optical switching strongly changes the shape of a Raman spectrum for initial GST film (Fig. S1, a). This difference in the shape is associated with a shift in the position of the peaks and a change in their mutual intensity, as well as with a decrease in the half-width at half maximum of all deconvoluted peaks. The decrease in the half-width and the shifts of peak positions can be explained by ordering the material[11] and mechanical stresses that may appearing in the volume of an amorphous material during its crystallization.[12, 13] The changes of spectrum profile are in good agreement with previously reported data and characteristic of the phase transition from the amorphous to the metastable cubic crystalline GST phase.[14, 15]


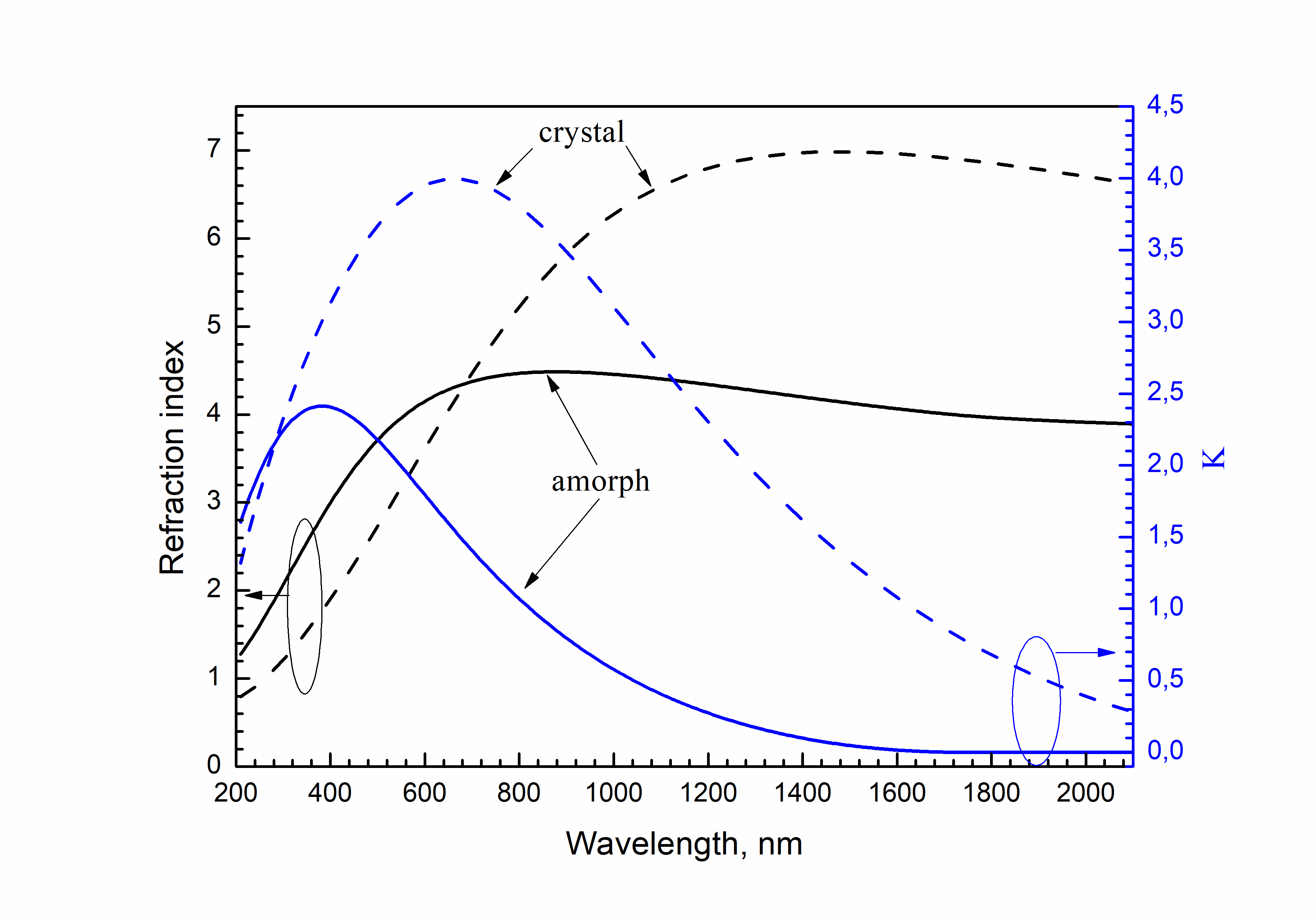


**Figure S2**: Dispersion of the refractive index and extinction coefficient for two different GST phases

The spectroscopic ellipsometer (HORIBA UVISEL 2) was used to determine the spectral dependences of the refractive index and extinction coefficient for the GST thin films in the wavelength range of 210 nm (5.9 eV)–2100 nm (0.59 eV). The spot size was 1 mm2. Measured spectra were evaluated by a Psi Delta program using a five-layered model (air–surface–GST– Si native oxide – Si substrate). A single Tauc–Lorentz (TL) oscillator was applied to obtain the optical properties of the films.

To estimate the refraction index (n) and the extinction coefficient (k) of the crystalline state, the as-deposited amorphous thin films were crystallized using the heating stage (Linkam HFS600E-PB4) at the temperature of 250 C for 30 min in the argon flow. The parameters of annealing treatment were selected considering the data of our previous research. The structure of the crystallized film was checked by X-ray diffraction (XRD, Rigaku SmartLab) and Raman spectroscopy (HORIBA LabRAM)


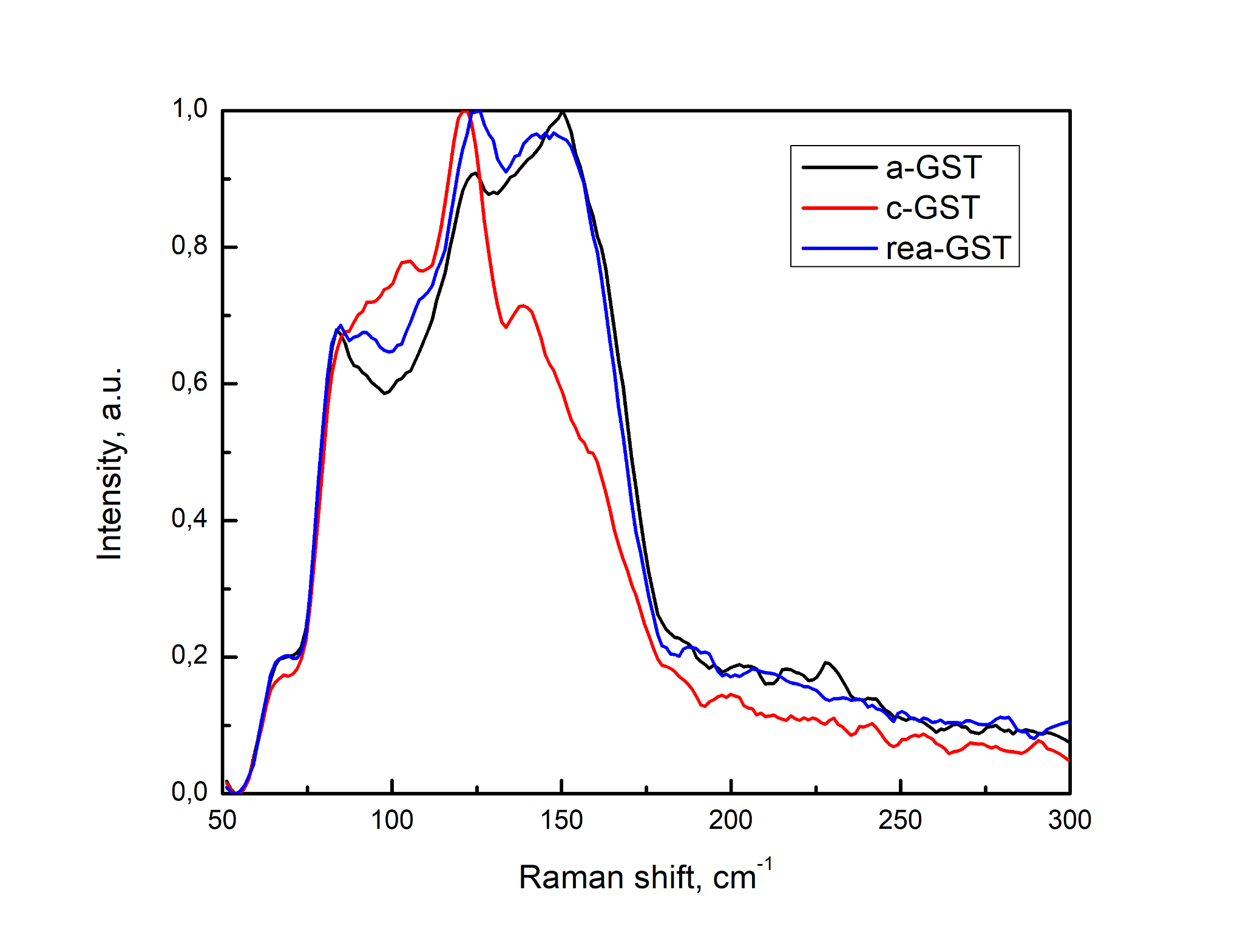


**Figure S3:** Raman spectra from three different state initial amorphous state (a-GST, black curved), crystalline state (c-GST, red curve), reamorphous state (rea-GST, blue curved)

We acquired Raman spectra using an excitation laser with 632 nm wavelength focused onto the spot of 1 μm in diameter. The excitation power was limited to 0.2 mW to avoid phase transformation by heating effect during the acquisition of spectra.

Figure 4S shows the ratio of the intensities of the 5th and 7th harmonics from the GST crystalline phase to the amorphous phase depending on the thickness of the GST film. On the one hand, it can be seen that the greatest contrast in harmonic intensities is observed for a thickness of 15 nm. On the other hand, at a thickness of 15 nm, it is impossible to guarantee the absence of a Ge oxide layer, as well as stable phase switching. Also, at a thickness of 15 nm, we failed to observe the 9th harmonic, since we could not use a high intensity of the pump laser, and as the intensity increased, the GST film was destroyed. The figure also shows that with a thickness of 50 nm, there is practically no difference in the intensities of harmonics from different phases. This is due to the fact that the absorption in the GST films increases significantly. The use of thicker GST layers does not make practical sense, since at thicknesses greater than 50 nm, there are already difficulties with stable switching of the GST phase, especially from the crystalline phase to the amorphous one.


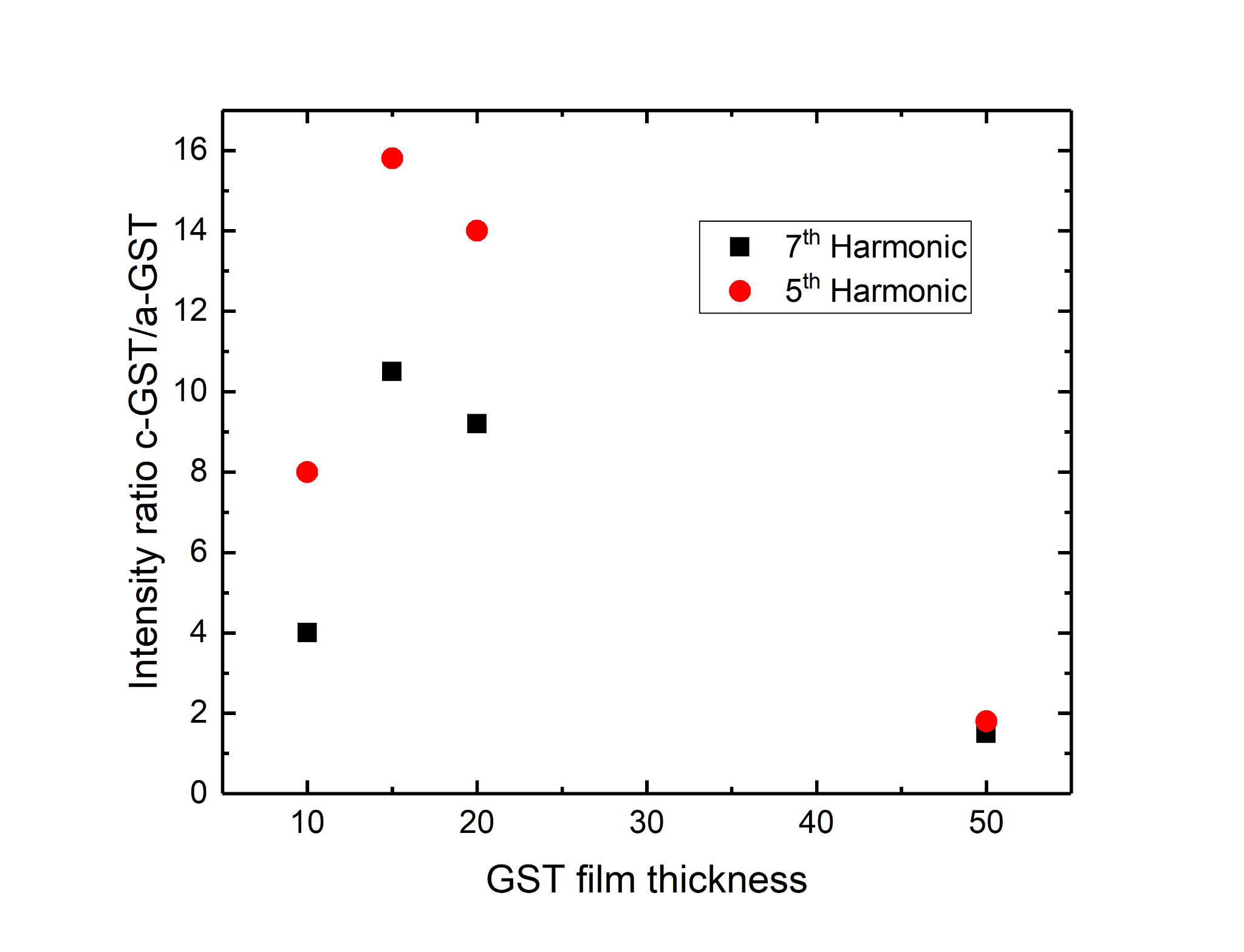


**Figure S4:** Ratio of the intensities of the 5th and 7th harmonics from the GST crystalline phase to the amorphous phase depending on the thickness of the GST film

**References**

[1] Němec P., Nazabal V., Moréac A., et al. “Amorphous and crystallized Ge–Sb–Te thin films deposited by pulsed laser: Local structure using Raman scattering spectroscopy” *Materials Chemistry and Physics*. Vol. 136, pp. 935-941, 2012. <https://doi.org/10.1016/j.matchemphys.2012.08.024>

[2] Andrikopoulos K. S., Yannopoulos S. N., Voyiatzis G. A., et al. “Raman scattering study of the a-GeTe structure and possible mechanism for the amorphous to crystal transition” *Journal of physics: condensed matter*. Vol. 18(3), pp. 965 2006. **DOI** 10.1088/0953-8984/18/3/014

[3] Shportko K., Revutska L., Paiuk O., et al. “Compositional dependencies in the vibrational properties of amorphous Ge-As-Se and Ge-Sb-Te chalcogenide alloys studied by Raman spectroscopy” *Optical Materials*. Vol. 73, pp. 489-496, 2017. <https://doi.org/10.1016/j.optmat.2017.08.042>

[4] Andrikopoulos K. S., Yannopoulos S. N., Kolobov A. V., et al. “Raman scattering study of GeTe and Ge2Sb2Te5 phase-change materials” *Journal of Physics and Chemistry of Solids*. Vol. 68(5-6), pp. 1074-1078, 2007. <https://doi.org/10.1016/j.jpcs.2007.02.027>

[5] Sahu S., Pandey S. K., Manivannan A., et al. “Direct evidence for phase transition in thin Ge1Sb4Te7 films using in situ UV–Vis–NIR spectroscopy and Raman scattering studies” *physica status solidi (b)*. Vol. 253, pp. 1069-1075, 2016 <https://doi.org/10.1002/pssb.201552803>

[6] De Bastiani R., Carria E., Gibilisco S., et al. “Ion-irradiation-induced selective bond rearrangements in amorphous GeTe thin films” *Physical Review B*. Vol.80, pp. 245205, 2009 10.1103/PhysRevB.80.245205

[7] Yang F., Xu L., Chen J., et al. “Nanoscale multilevel switching in Ge2Sb2Te5 thin film with conductive atomic force microscopy” *Nanotechnology*. Vol. 27, pp. 035706, 2015 10.1088/0957-4484/27/3/035706

[8] Xu J., Qi C., Chen L., et al. “The microstructural changes of Ge2Sb2Te5 thin film during crystallization process” *AIP Advances*. Vol. 8, pp. 055006, 2018 <https://doi.org/10.1063/1.5025204>

[9] Bouška M., Pechev S., Simon Q., et al. “Pulsed laser deposited GeTe-rich GeTe-Sb2Te3 thin films” *Scientific Reports*. Vol. 6, pp. 1-10, 2016 <https://doi.org/10.1038/srep26552>

[10] Sosso, G. C., Caravati, S., Mazzarello, R., Bernasconi, M. “Raman spectra of cubic and amorphous Ge Sb Te from first principles” *Physical Review B*. Vol. 83, pp. 134201, 2011 10.1103/PhysRevB.83.134201

[11] Shportko K. V. “Disorder and compositional dependences in Urbach-Martienssen tails in amorphous (GeTe)x(Sb2Te3)1−x alloys” *Scientific reports*. Vol. 9, pp. 6030, 2019 <https://doi.org/10.1038/s41598-019-42634-8>

[12] Zhu Z., Liu F. R., Wang Z. et al. “Comparative study on crystallization characteristics of amorphous Ge2Sb2Te5 films by an ultraviolet laser radiation and isothermal annealing” *Applied Surface Science*. Vol. 335, pp. 184-188, 2015 <https://doi.org/10.1016/j.apsusc.2015.02.052>

[13] De Wolf I. *Semiconductor science and technology*. **1996** *11*, 139

[14] Němec P., Moreac A., Nazabal V., et al. “Ge–Sb–Te thin films deposited by pulsed laser: An ellipsometry and Raman scattering spectroscopy study” *Journal of Applied Physics*. Vol. 106, pp. 103509, 2009 <https://doi.org/10.1063/1.3259435>

[15] Gu T., Wang J., Liu H., Wang Z., Luo Y., Liu P., Zhong J., Wang G. “One-step phase transition and thermal stability improvement of Ge2Sb2Te5 films by erbium-doping” *Vacuum*. Vol. 145, pp. 258-261, 2017. <https://doi.org/10.1016/j.vacuum.2017.09.008>
